# Prognostic Value of Dynamic FDG PET-Derived Myocardial Glucose Metabolism in Ischemic Cardiomyopathy with Supportive External Gene-Expression Analysis

**DOI:** 10.3390/diagnostics16142237

**Published:** 2026-07-17

**Authors:** Kuan-Yin Ko, Shan-Ying Wang, Hao-Yuan Tsai, Chien-Lin Lee, Jung-Cheng Hsu, Chung-Ming Tu, Yu-Chien Shiau, Kuan-Ming Chiu, Wen-Pin Chen, Yen-Wen Wu

**Affiliations:** 1 Department of Nuclear Medicine, National Taiwan University Cancer Center and College of Medicine, National Taiwan University, Taipei 100233, Taiwan; 2 Department of Nuclear Medicine, Far Eastern Memorial Hospital, New Taipei City 220216, Taiwan; 3 Division of Cardiology, Cardiovascular Medical Center, Far Eastern Memorial Hospital, New Taipei City 220216, Taiwan; 4 Division of Cardiovascular Surgery, Cardiovascular Medical Center, Far Eastern Memorial Hospital, New Taipei City 220216, Taiwan; 5 Institute of Pharmacology, College of Medicine, National Taiwan University, Taipei 100233, Taiwan; 6 School of Medicine, National Yang Ming Chao Tung University, Taipei 112304, Taiwan

**Keywords:** dynamic FDG PET, myocardial glucose utilization, transcriptomics, prognosis, ischemic cardiomyopathy

## Abstract

**Background/Objectives:** To evaluate whether global myocardial glucose metabolism and its regional heterogeneity, quantified using dynamic ^18^F-fluorodeoxyglucose positron emission tomography (FDG PET), are associated with cardiovascular outcomes in patients with ischemic cardiomyopathy. **Methods:** The analytic cohort included 120 patients with suspected ischemic cardiomyopathy who underwent thallium-201 perfusion single photon emission tomography and dynamic FDG PET. Global myocardial metabolic rate of glucose (MRGlu) and coefficient of variation in FDG uptake were calculated from scar-excluded myocardium. Major adverse cardiac events (MACEs) were assessed over a median follow-up of 2.5 years. Public myocardial gene-expression datasets (GSE116250 and GSE135055) were analyzed to explore glucose metabolism-related transcriptional patterns relevant to the imaging findings. **Results:** Fifty-one patients (42.5%) experienced MACEs. Lower global MRGlu was independently associated with improved MACE-free survival (hazard ratio 0.48, 95% confidence interval 0.27–0.87). Among patients with low global MRGlu, a high coefficient of variation predicted favorable outcomes (hazard ratio 0.35, 95% confidence interval 0.18–0.68), indicating beneficial metabolic heterogeneity. Analysis of external gene-expression data demonstrated consistent downregulation of glucose-handling genes in end-stage heart failure across ischemic and dilated etiologies. A higher expression of SLC2A1, HK1, IRS1, and PKM was associated with faster progression to transplantation. **Conclusions:** In ischemic cardiomyopathy, dynamic FDG PET-derived quantification of myocardial glucose metabolism provides prognostic information beyond traditional viability assessment. Low global metabolism combined with preserved metabolic heterogeneity helps identify patients with favorable clinical outcomes.

## 1. Introduction

Coronary artery disease and its associated metabolic disorders remain major contributors to global morbidity and mortality [1]. Cardiac metabolism undergoes profound reprogramming under pathological stress, including ischemic cardiomyopathy. Although an acute increase in glucose utilization may represent an adaptive mechanism to maintain myocardial energy supply during ischemia, persistent activation of glycolytic pathways can promote maladaptation, contractile dysfunction, and progression to heart failure. In addition, metabolic regulation of growth, proliferation, and survival pathways is increasingly recognized as a key determinant of cardiac biology [2,3].

Clinically, assessment of myocardial viability using a combination of perfusion and ^18^F-fluorodeoxyglucose (FDG) uptake has become an established tool for identifying dysfunctional but viable myocardium with potential for functional recovery following revascularization. This viability information has important implications in selecting patients who may benefit from invasive coronary interventions [4,5,6]. However, current therapeutic strategies for heart failure continue to focus predominantly on improving perfusion, reducing hemodynamic load, and suppressing inflammation, with relatively limited emphasis on metabolic remodeling. A more refined understanding of myocardial metabolic changes could provide opportunities to develop personalized and precise treatment strategies.

Dynamic cardiac positron emission tomography (PET) enables quantitative evaluation of myocardial glucose metabolism and its spatial heterogeneity, providing a window into metabolic remodeling in vivo [7,8]. However, the prognostic relevance of FDG-derived metabolic indices in ischemic cardiomyopathy remains insufficiently defined. Therefore, in this study, we sought to integrate dynamic FDG PET-derived quantification of global myocardial glucose metabolism and metabolic heterogeneity into viability assessment and to investigate their prognostic relevance in patients with ischemic cardiomyopathy. To further contextualize and corroborate these findings, we performed external transcriptomic analyses using publicly available datasets.

## 2. Methods

### 2.1. Study Population

We established a cohort of consecutive patients with suspected ischemic cardiomyopathy who underwent electrocardiography (ECG)-gated thallium-201 (Tl-201) single-photon emission computed tomography (SPECT) and dynamic FDG PET between November 2012 and January 2020. Patients were referred prior to coronary intervention or heart transplantation, with revascularization decisions made by treating physicians based on imaging, clinical factors, and patient preference. Patients with severe valvular disease, idiopathic dilated cardiomyopathy (DCM), or a life expectancy of <3 months due to catastrophic illness were excluded. We also excluded patients who underwent revascularization procedures and those who experienced myocardial infarction or acute coronary syndrome during their cardiac SPECT and PET studies. A total of 130 patients with suspected ischemic cardiomyopathy who underwent Tl-201 SPECT and dynamic FDG PET between November 2012 and January 2020 were initially identified. Ten patients were excluded because of inadequate image quality (*n* = 5), reconstruction error (*n* = 1), or unavailable original imaging data (*n* = 4). The final analytic cohort therefore included 120 patients (Figure S1). Among them, 79 patients (65.8%) had been included in our earlier publication, which investigated the quantification of myocardial glucose uptake across different perfusion–metabolism patterns and its clinical significance [9]. The current expanded cohort analysis focused on global myocardial glucose metabolism, excluding scarred regions, and metabolic heterogeneity to evaluate their prognostic relevance. Informed consent was obtained from all participants, and the study protocol was approved by the Ethics Committee of Far Eastern Memorial Hospital (IRB No. FEMH-IRB-101037-F).

### 2.2. Tl-201 Cardiac SPECT and Dynamic FDG PET/CT

Cardiac SPECT and dynamic FDG PET/CT imaging were performed using a cadmium-zinc-telluride camera (Discovery NM530c, GE Healthcare, Chicago, IL, USA) and a PET/CT scanner (GE Discovery VCT GE Medical Systems, Milwaukee, WI, USA). A complete description of the procedure and scan protocol, as well as details of the quantification of myocardial glucose utilization, have been reported previously [9,10]. In brief, patients underwent dipyridamole stress (0.56 mg/kg) and redistribution Tl-201imaging (2–3 mCi, 74–111 MBq), or rest-redistribution imaging if stress testing was contraindicated. Functional data, including left ventricular ejection fraction (LVEF), were further generated using Quantitative Perfusion SPECT/Quantitative Gated SPECT (QPS/QGS) software (v4.0, Cedars-Sinai Medical Center, Los Angeles, CA, USA). Before undergoing dynamic FDG PET/CT (10 mCi, 370 MBq), an oral glucose load was administered to patients with no diabetes, whereas insulin was given intravenously to patients with diabetes according to their measured blood glucose levels [11,12,13]. The dynamic FDG PET protocol consisted of a 50 min list-mode acquisition, which was rebinned into 32 dynamic frames: 12 × 10 s, 6 × 20 s, 6 × 60 s, and 8 × 300 s. The final 20 min of list-mode data were used to generate ECG-gated images. All PET images were reconstructed using ordered-subsets expectation maximization with 20 subsets and 3 iterations. The in-plane spatial resolution was 6 mm full width at half maximum, and the reconstruction matrix was 128 × 128 pixels. Static Tl-201 redistribution SPECT was used to assess relative myocardial perfusion and was combined with FDG PET metabolic imaging to define perfusion–metabolism patterns. Each myocardial segment was visually scored on a 5-point scale from normal (0) to absent uptake [4] using short- and long-axis images and corresponding polar maps [14]. Segments were categorized as mismatch (reduced perfusion with relatively preserved glucose metabolism) or match (concordant reduction in both perfusion and metabolism). Summed rest scores and summed FDG scores were derived by adding the respective segmental scores across the standard 17-segment model. Corresponding summed rest scores and FDG scores for matched and mismatched segments were also calculated.

Quantitative image analysis was performed using PMOD software, version 3.7 (PMOD Technologies, Zurich, Switzerland). Dynamic PET images were reoriented into standard cardiac short-axis, vertical long-axis, and horizontal long-axis views. For the generation of the image-derived blood input function, the left ventricular (LV) cavity volume of interest was delineated semi-automatically using endocardial and epicardial contours in the three cardiac projections. Manual adjustments were performed when necessary to ensure that the LV-cavity volume of interest remained within the blood pool and avoided spillover from the myocardium, papillary muscles, or adjacent structures. The resulting LV-cavity time–activity curve was used as the image-derived blood input function. Myocardial regions of interest were assigned according to the standard 17-segment model. Segmental myocardial time–activity curves were generated from the dynamic PET data and fitted using Patlak graphical analysis. The Patlak slope was used to estimate the net FDG influx constant, and the myocardial metabolic rate of glucose was calculated as:

MRGlu = Patlak slope × plasma glucose concentration/lumped constant,

using a lumped constant of 0.67, with unit conversion to μmol/min/100 g. Global MRGlu was calculated as the average of segmental MRGlu values across all evaluable myocardial segments after excluding matched perfusion–metabolism defect segments. Matched segments were defined as segments showing concordantly reduced Tl-201 redistribution and FDG uptake and were excluded because they represented scarred/nonviable myocardium. To assess intra-myocardial heterogeneity, the coefficient of variation (CoV) was calculated across all evaluable segments, again excluding matched regions. CoV was defined as the standard deviation of segmental FDG values divided by their mean and expressed as a percentage [15]. Figure S2 illustrates the four representative metabolic patterns defined by combinations of MRGlu and CoV.

### 2.3. Patient Follow-Up

All patients were followed clinically for at least 6 months after the SPECT/PET studies. Major adverse cardiac events (MACEs) included cardiac and non-cardiac death, hospitalization for acute myocardial infarction, unplanned or late (more than 6 months after the SPECT/PET studies) coronary intervention, implantation of a left ventricular assist device or implantable cardioverter-defibrillator, and hospitalization for heart failure. Data on clinical parameters, laboratory findings, MACEs, and coronary interventions were reviewed from the hospital’s electronic medical records. MACE-free survival was calculated from the date of the FDG PET study to the occurrence of the first MACE.

### 2.4. External Gene-Expression Dataset Analysis

We analyzed publicly available myocardial gene-expression datasets derived from RNA sequencing from human left ventricular myocardium obtained from the Gene Expression Omnibus (GEO) (http://www.ncbi.nlm.nih.gov/geo accessed on 1 October 2025) dataset GSE116250. This dataset includes samples from non-failing donor controls, patients with ischemic cardiomyopathy, and patients with DCM. All non-failing donor controls, ischemic cardiomyopathy, and DCM samples with complete expression data were included. The expression matrix was downloaded in transcript per million mapped reads (RPKM)-normalized format, as provided by the original investigators. We selected 10 genes central to myocardial glucose uptake and utilization, based on their established roles in glycolysis, insulin signaling, and metabolic regulation: *AKT1*, *HK1*, *HK2*, *INSR*, *IRS1*, *PFKM*, *PKM*, *PRKAA1*, *SLC2A1 (GLUT1)*, and *SLC2A4 (GLUT4)*. These genes were chosen to align with mechanistic hypotheses generated from our FDG-PET cohort. This hypothesis-driven analysis focused on selected genes related to myocardial glucose metabolism and was not a genome-wide transcriptomic analysis.

As prognosis-linked transcriptomic datasets specific to ischemic cardiomyopathy are not available, we evaluated the prognostic relevance of the glucose-metabolic pattern identified in our ischemic cardiomyopathy PET cohort using a DCM prognosis dataset as a surrogate (GSE135055) [16]. This approach is supported by our analysis of GSE116250, in which glucose-metabolism genes exhibited similar directional changes in ischemic cardiomyopathy and DCM compared with non-failing controls, with no major opposing effects. Prior literature also supports convergence of metabolic remodeling across heart failure etiologies, providing biological justification for applying DCM data to explore the potential prognostic relevance of this pathway [17,18,19]. This dataset included myocardial samples from 21 patients with heart failure, along with detailed survival information (time to heart transplantation). For analysis, patients were classified as fast progression or slow progression according to the median time to transplantation. To capture the overall expression pattern of glucose metabolism-related genes significantly associated with prognosis, we constructed a composite gene score. For each gene, expression values were standardized across all patients by calculating z-scores:

(1) $$z_{ij}=\frac{x_{ij}-{\bar{x}}_{j}}{\sigma_{j}}$$

where $x_{ij}$ is the expression level of gene j in patient i, ${\bar{x}}_{j}$ is the mean expression of gene j across all patients, and $\sigma_{j}$ is the corresponding standard deviation. The gene-specific z-scores for each patient were then summed without weighting to generate the composite gene score.

### 2.5. Statistical Analysis

Continuous data are expressed as mean ± standard deviation, and categorical variables as frequency distribution. Differences between two groups were analyzed using Student’s *t* test or Mann–Whitney U test for continuous variables and the chi-square test or Fisher’s exact test for categorical variables. A univariate Cox proportional hazards model was used to test associations between clinical outcomes and variables. Multivariate Cox regression analyses, adjusting for potentially relevant variables, were performed to identify independent predictors of outcome. Survival curves were generated using the Kaplan–Meier method and compared using the log-rank test. All tests were two-tailed, and a *p* value < 0.05 was considered statistically significant. Analyses were performed using MedCalc statistical software (version 12.7, Ostend, Belgium). Graphics were generated primarily using MedCalc and Python (version 3.13).

## 3. Results

### 3.1. Dynamic FDG PET Cohort

A total of 120 patients were included in this study, and the mean follow-up duration was 2.5 ± 2.1 (0.5–8.4) years. The average time between the date of FDG PET and cardiac SPECT was 7.8 ± 8.8 (0–31) days. Fifty-one (42.5%) patients experienced MACEs, including death (*n =* 22), acute myocardial infarction (*n =* 4), late coronary intervention (*n =* 12), device implantation (*n =* 2), and heart failure (*n =* 11). Table 1 and Table 2 show the clinical characteristics, results of myocardial viability test, and MRGlu values of the patients with and without MACEs. Compared with the MACE group, the non-MACE group had a higher proportion of patients receiving statin therapy (68.1 vs. 54.9%, *p* = 0.02). No statistically significant differences were observed between groups in terms of clinical cardiovascular risk factors, LVEF, SPECT-derived ventricular volumes, or myocardial viability findings. Following the myocardial viability test, 26 of the 120 patients underwent revascularization with either percutaneous coronary intervention or coronary artery bypass graft surgery. Of these 26 patients, 12 (23.5%) experienced MACEs, whereas 14 (20.3%) did not.

**Figure 1:**
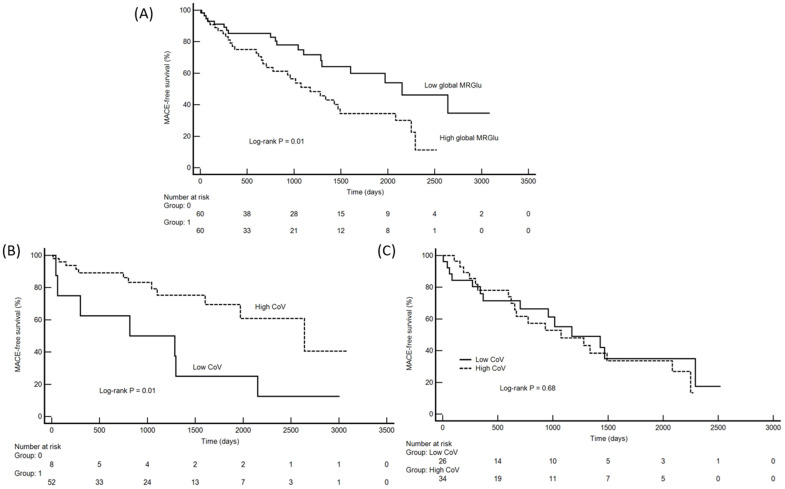
Kaplan–Meier curves for MACE-free survival stratified by myocardial glucose metabolism and metabolic heterogeneity. (**A**) Overall survival curves comparing low versus high MRGlu, categorized by the median value. (**B**) Among patients with low MRGlu, further stratification by metabolic heterogeneity using CoV (high vs. low, based on the median). (**C**) Among patients with high MRGlu, corresponding stratification by CoV (high vs. low, based on median). Abbreviations: MACE, major adverse cardiovascular event; CoV, coefficient of variation; MRGlu, myocardial metabolic rate of glucose.

**Figure 2:**
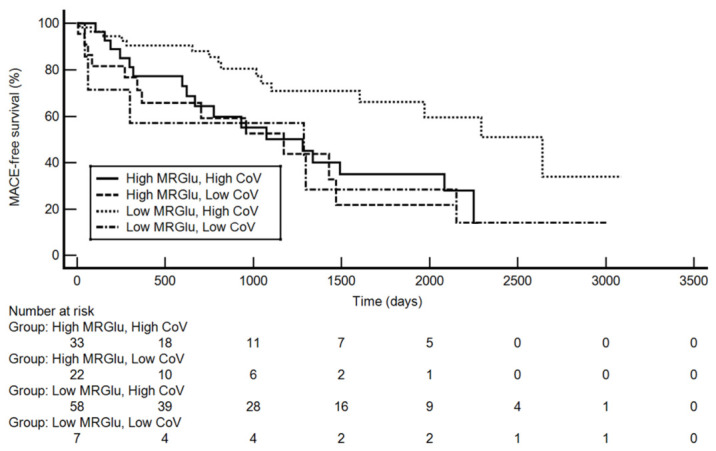
Kaplan–Meier curves for MACE-free survival stratified into four metabolic phenotypes defined by global MRGlu and CoV: high MRGlu/high CoV, high MRGlu/low CoV, low MRGlu/high CoV, and low MRGlu/low CoV. The number-at-risk table below the plot indicates the number of patients remaining under observation and free of MACE at each specified follow-up time point. Abbreviations: MACE, major adverse cardiovascular event; CoV, coefficient of variation; MRGlu, myocardial metabolic rate of glucose.

Patients in the non-MACE group exhibited lower global MRGlu values compared with those in the MACE group (22.3 ± 16.2 vs. 32.3 ± 23.4 μmol/min/100 g, *p* = 0.006). When analyzed as a categorical variable using the median cutoff, lower global MRGlu was likewise associated with the non-MACE group. In addition, the proportion of patients with high CoV (above the median cutoff) was greater in the non-MACE group. In multivariable Cox regression analysis (Table 3), higher global MRGlu as a continuous variable was independently associated with adverse outcomes (hazard ratio [HR] 1.01, 95% confidence interval [CI] 1.002–1.02, *p* = 0.009). When dichotomized by the median cutoff, patients with low global MRGlu demonstrated a significantly lower risk of MACE (HR 0.48, 95% CI 0.27–0.87, *p* = 0.01). High CoV, when analyzed alone as a categorical variable, was not significantly associated with outcomes (HR 0.65, 95% CI 0.35–1.21, *p* = 0.18). In a joint model including both continuous and categorical measures of global MRGlu, the categorical variable (low vs. high) remained significant (HR 0.48, 95% CI 0.27–0.87, *p* = 0.01). A significant interaction was also observed between high CoV and low global MRGlu (HR 0.35, 95% CI 0.18–0.68, *p* = 0.001). Among patients with low global MRGlu, those with high CoV exhibited longer MACE-free survival compared with those with low CoV, whereas no such difference was observed among patients with high global MRGlu (Figure 1 and Figure 2).

### 3.2. Public Gene Expression Data Analysis

**Figure 3:**
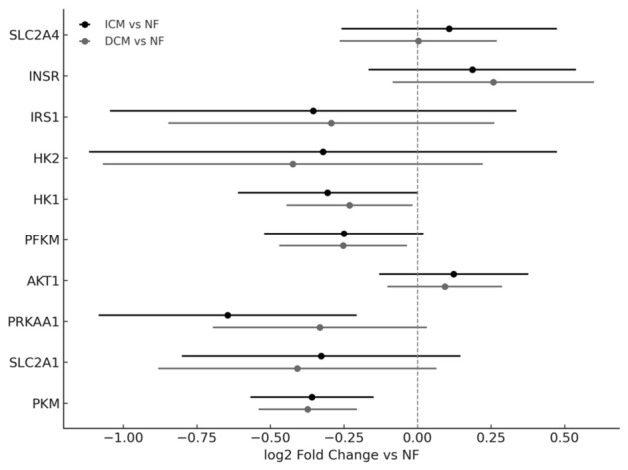
Differential expression of glucose-handling genes in ischemic and dilated cardiomyopathy. Forest plot showing log_2_ fold change (±95% confidence interval) of key myocardial glucose-metabolism genes in ischemic cardiomyopathy (ICM; black) and dilated cardiomyopathy (DCM; gray) compared with non-failing hearts (NF). Abbreviations: AKT1, AKT serine/threonine kinase 1; DCM, dilated cardiomyopathy; HK1, hexokinase 1; HK2, hexokinase 2; ICM, ischemic cardiomyopathy; INSR, insulin receptor; IRS1, insulin receptor substrate 1; NF, non-failing heart; PFKM, phosphofructokinase, muscle type; PKM, pyruvate kinase M1/2; PRKAA1, protein kinase AMP-activated catalytic subunit alpha 1 (AMPKα1); SLC2A1, solute carrier family 2 member 1 (GLUT1); SLC2A4, solute carrier family 2 member 4 (GLUT4).

In the discovery cohort (GSE116250), glucose-metabolism-related genes demonstrated consistent directional changes in both ischemic cardiomyopathy and DCM compared with controls, with no evidence of large opposing effects between the two etiologies (Figure 3). *PKM* (ischemic cardiomyopathy −0.36; DCM −0.37 log_2_FC) and *HK1* (ischemic cardiomyopathy −0.31; DCM −0.23 log_2_FC) were significantly downregulated in both etiologies. *PFKM* was significantly reduced in DCM and showed a borderline decrease in ischemic cardiomyopathy, while *PRKAA1* (*AMPKα1*) was strongly suppressed in ischemic cardiomyopathy (*p* = 0.0004) and modestly reduced in DCM with borderline significance. *SLC2A1* (*GLUT1*) also demonstrated a consistent downward trend across groups, although not statistically significant. Additional glycolytic regulators, *HK2* and *IRS1*, were numerically lower in both ischemic cardiomyopathy (−0.32 and −0.35 log_2_FC) and DCM (−0.42 and −0.29 log_2_FC), but neither reached statistical significance. In contrast, *INSR* and *AKT1* exhibited modest positive log_2_FC values in both ischemic cardiomyopathy (*INSR* +0.19; *AKT1* +0.12) and DCM (*INSR* +0.26; *AKT1* +0.09), although their confidence intervals crossed zero. *SLC2A4* (*GLUT4*) expression remained largely unchanged. Overall, these findings indicate that key glycolytic enzymes (PKM, PFKM, HK1, and PRKAA1) and the glucose transporter GLUT1 are downregulated in both ischemic cardiomyopathy and DCM.

**Figure 4:**
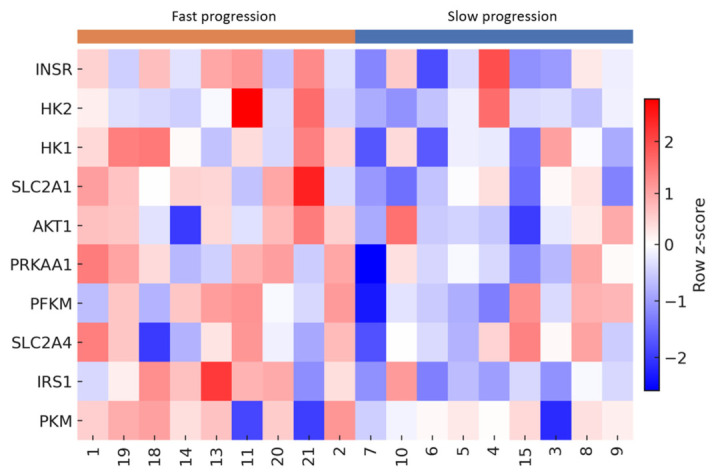
Heatmap of glucose-related gene expression in slow- and fast-progression heart failure. Each column represents a patient (numbers indicate sample ID), with the top horizontal bar denoting clinical group: blue for slow progression and orange for fast progression. Each row corresponds to a glucose metabolism-related gene. Values are shown as per-gene z-scores, with red indicating expression above the mean and blue indicating expression below the mean for that gene across all patients. Abbreviations: AKT1, AKT serine/threonine kinase 1; HK1, hexokinase 1; HK2, hexokinase 2; INSR, insulin receptor; IRS1, insulin receptor substrate 1; PFKM, phosphofructokinase, muscle type; PKM, pyruvate kinase M1/2; PRKAA1, protein kinase AMP-activated catalytic subunit alpha 1 (AMPKα1); SLC2A1, solute carrier family 2 member 1 (GLUT1); SLC2A4, solute carrier family 2 member 4 (GLUT4).

**Figure 5:**
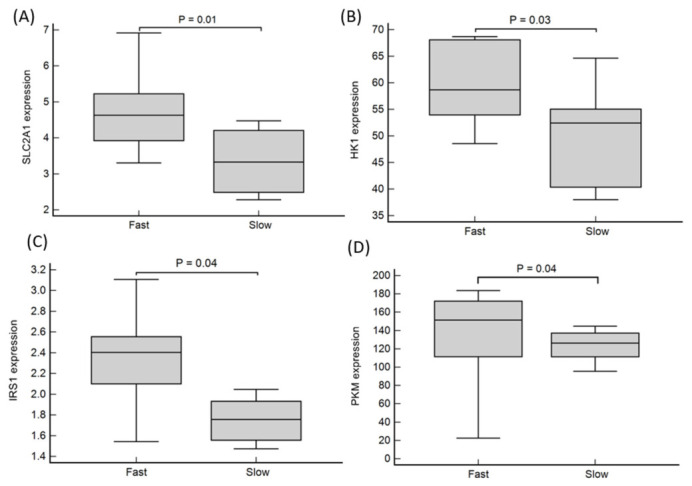
Expression of SLC2A1 (**A**), HK1 (**B**), IRS1 (**C**), and PKM (**D**) in slow- and fast-progression heart failure. Box–whisker plots showing expression levels in patients with slow progression (*n* = 9) and fast progression (*n* = 9) of heart failure. Boxes indicate the interquartile range (IQR) with the median indicated as a horizontal line; whiskers extend to the minimum and maximum observed values. The connector line with *p* value represents the result of the Mann–Whitney U test. Abbreviations: HK1, hexokinase 1; IRS1, insulin receptor substrate 1; PKM, pyruvate kinase M1/2; SLC2A1, solute carrier family 2 member 1 (GLUT1).

**Figure 6:**
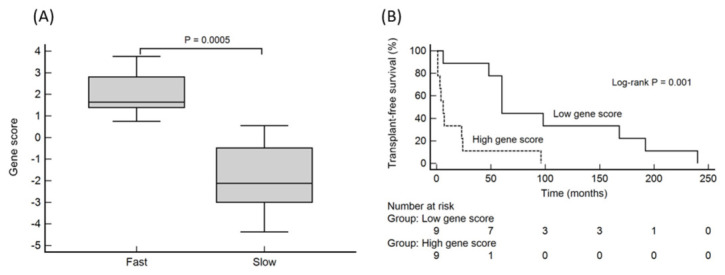
(**A**) Box–whisker plot of the composite 4-gene score in fast- and slow-progression heart failure. (**B**) Transplant-free survival stratified by high versus low composite 4-gene scores.

As prognosis-linked transcriptomic datasets for ischemic cardiomyopathy are lacking, we used a DCM prognosis dataset (GSE135055) as a surrogate to assess the clinical relevance of the glucose-metabolic program observed in our ischemic cardiomyopathy PET cohort. Figure 4 depicts the heatmap of glucose-related gene expression stratified by clinical progression status. In this cohort, patients with slow progression of heart failure exhibited significantly lower expression of *SLC2A1*, *HK1*, *IRS1*, and *PKM* compared with those with fast progression (Figure 5; all *p* < 0.05). A composite gene score derived from these four transcripts was also reduced in the slow-progression group and was associated with prolonged transplant-free survival (Figure 6). Kaplan–Meier analyses of the four transcripts, dichotomized by median expression, are shown in Figure S3.

## 4. Discussion

In this study, dynamic FDG PET was incorporated into viability testing to quantify myocardial glucose metabolism. In patients with ischemic cardiomyopathy, higher global glucose metabolism within scar-excluded myocardium was associated with poorer cardiovascular outcomes. Stratification by the CoV of FDG uptake in scar-excluded myocardium revealed an effect-modifying relationship: among patients with low global metabolism, a higher CoV, reflecting greater regional heterogeneity, was associated with more favorable outcomes, whereas CoV provided no prognostic benefit in patients with high global metabolism. Our analysis of the external gene-expression datasets showed that, in end-stage heart failure of both ischemic and dilated etiologies, genes involved in myocardial glucose handling were downregulated, suggesting a shared metabolic phenotype. Although not intended as direct validation, these datasets demonstrated a concordant pattern whereby higher expression of key glucose-metabolism genes (*SLC2A1*, *HK1*, *IRS1*, and *PKM*) tended to progress more rapidly to transplantation.

### 4.1. Dynamic FDG Cohort

In our cohort, statin therapy was associated with fewer MACEs, although it did not emerge as a statistically significant protective factor in Cox regression, consistent with prior evidence of statin benefit across heart failure phenotypes, irrespective of underlying etiology or LVEF [17,18].

Our study demonstrated that patients with low global MRGlu and high CoV experienced fewer MACE events. In multivariable Cox regression, low global MRGlu emerged as an independent protective factor. Stratified analyses further revealed that among patients with low global MRGlu, those with high CoV had more favorable cardiovascular outcomes, whereas no such prognostic difference was observed in the high MRGlu subgroup. From a pathophysiological standpoint, chronic ischemic heart disease is characterized by severely impaired oxygen supply. Under such conditions, a metabolic shift from fatty acid oxidation to glucose utilization enhances oxygen efficiency for adenosine triphosphate (ATP) synthesis. However, reliance on glycolysis, with reduced ATP yield from oxidative metabolism, may contribute to progressive energy starvation and the development of chronic heart failure [20,21,22]. Within this framework, patients exhibiting low myocardial glucose utilization, but high CoV, may reflect preserved heterogeneity and flexibility of myocardial substrate metabolism. Such metabolic adaptability could mitigate energy deficiency and, in turn, translate into improved clinical outcomes.

### 4.2. GEO Datasets

Table S1 summarizes the pathway classification and established biological role of each gene selected for the hypothesis-driven transcriptomic analysis [23,24,25,26,27,28,29,30,31]. Although acute ischemic or hypoxic stress promotes a compensatory shift toward glycolysis to maintain ATP under low oxygen conditions, the transcriptomic data demonstrated that in chronic, end-stage heart failure, regardless of etiology, glucose-uptake and glycolytic genes (*GLUT1*, *HK1*, *PFKM*, *PKM*, and *PRKAA1*) are downregulated. This reflects a transition from adaptive short-term metabolic reprogramming to maladaptive chronic suppression of glucose metabolism, consistent with impaired insulin signaling, pyruvate dehydrogenase (PDH) inhibition, and increased reliance on alternative fuels. Large-scale metabolomic studies in failing human hearts have demonstrated reduced glucose utilization accompanied by increased uptake of ketone bodies and lactate [32,33], while transcriptomic analyses consistently show downregulation of carbohydrate-metabolism genes in advanced heart failure [2,34]. In parallel, impaired insulin signaling and PDH inhibition, mediated in part via pyruvate dehydrogenase kinase 4 (PDK4), have been identified as major mechanisms uncoupling glycolysis from glucose oxidation [35,36]. Thus, our findings align with prior evidence that the failing human heart ultimately exhibits reduced glucose utilization despite initial stress-induced upregulation.

Consistent with our FDG PET findings, higher expression of four key glucose-metabolism genes in heart failure was associated with shorter transplant-free survival. *SLC2A1*, encoding GLUT1, facilitates basal glucose transport, whereas IRS1 serves as a key adaptor in insulin signaling, promoting GLUT4 translocation and insulin-dependent glucose uptake. Following entry into the cardiomyocyte, glucose is phosphorylated by HK1 to glucose-6-phosphate, effectively trapping it intracellularly and directly influencing FDG retention on PET imaging. PKM, a pivotal glycolytic enzyme, catalyzes the terminal step of glycolysis and determines downstream energy yield. Together, these transcripts represent sequential checkpoints encompassing glucose entry, intracellular trapping, insulin responsiveness, and glycolytic utilization. Reduced expression across this axis would be expected to constrain myocardial glucose uptake [37,38,39]. These findings suggest that transcriptional regulation of myocardial glucose metabolism may help explain the adverse prognostic implications of heightened FDG uptake observed in heart failure.

### 4.3. Limitations

Some limitations of this study should be acknowledged. First, this is an observational cohort study with a small sample size, limiting generalizability. Second, Myocardial perfusion was assessed using static Tl-201 SPECT rather than PET perfusion imaging. Therefore, perfusion was evaluated on a relative basis, and absolute myocardial blood flow was not quantified. Although standardized acquisition, reconstruction, and image interpretation were applied, the intrinsic limitations of Tl-201 SPECT, including potential underestimation of perfusion defects, could not be completely eliminated and may have influenced the classification of perfusion–metabolism patterns. Third, myocardial fatty acid imaging was not performed, preventing a more comprehensive evaluation of cardiac metabolic profiles. In addition, changes in Taiwan’s Bureau of National Health Insurance reimbursement policies for dyslipidemia may partly explain the relatively low rate of statin use observed in our study. We did not analyze other factors affecting myocardial metabolism. Finally, the external gene-expression analysis was hypothesis-driven and limited to selected metabolism-related genes, rather than being a comprehensive transcriptomic or pathway-level analysis.

### 4.4. Conclusions

Quantification of myocardial glucose metabolism using dynamic FDG PET, when incorporated into viability assessment, may offer additional prognostic insights in patients with ischemic cardiomyopathy. Lower myocardial glucose metabolism was independently associated with more favorable outcomes, a finding supported by concordant patterns observed in external gene-expression datasets. Stratification by CoV further suggested that patients with low myocardial glucose metabolism and higher heterogeneity may experience fewer MACE events.

## Figures and Tables

**Table 1:** Patient characteristics.

Parameters	MACE (*n* = 51)	No MACE (*n* = 69)	*p*-Value
Age (years)	63.2 ± 12.6	62.2 ± 12.8	0.66
Female	7 (13.7)	5 (7.2)	0.35
Hypertension	37 (72.5)	50 (72.5)	1.0
Diabetes	25 (49.0)	42 (60.9)	0.26
Hyperlipidemia	29 (56.9)	40 (58.0)	1.0
Smoker	37 (72.5)	42 (60.9)	0.24
Prior MI	11 (21.56)	19 (27.53)	0.52
Prior PCI or CABG	20 (39.2)	29 (42.0)	0.85
Creatinine (mg/dL)	2.0 ± 2.16	1.71 ± 1.81	0.43
Medication			
Insulin	4 (7.8)	5 (7.2)	1.0
OHA	12 (23.5)	25 (46.2)	0.16
Statin	28 (54.9)	47 (68.1)	0.02
Beta-blocker	35 (68.6)	49 (71.0)	0.27
CCB	3 (5.8)	4 (5.8)	1.0
ACEI	23 (45.1)	23 (33.3)	0.44
ARB	9 (17.6)	10 (14.5)	1.0
Laboratory data			
HbA1c (%)	6.7 ± 1.3	7.5 ± 2.0	0.05
T-CHO (mg/dL)	168.4 ± 48.4	158.7 ± 42.4	0.34
TG (mg/dL)	150.7 ± 97.0	151.1 ± 99.5	0.98
LDL-C (mg/dL)	107.5 ± 43.7	101.7 ± 38.8	0.54
LVEF (%)	25.0 ± 9.2	25.2 ± 9.9	0.95
EDV (mL)	177.3 ± 60.5	181.1 ± 65.6	0.75
ESV (mL)	135.6 ± 55.4	141.1 ± 63.0	0.63

Abbreviations: ACEI, angiotensin-converting enzyme inhibitor; ARB, angiotensin II receptor blocker; CABG, coronary artery bypass grafting; CCB, calcium channel blocker; EDV, end-diastolic volume; ESV, end-systolic volume; HbA1c, hemoglobin A1c; LDL-C, low-density lipoprotein cholesterol; LVEF, left ventricular ejection fraction; MACE, major adverse cardiovascular events; MI, myocardial infarction; OHA, oral hypoglycemic agent; PCI, percutaneous coronary intervention; T-CHO, total cholesterol; TG, triglyceride.

**Table 2:** FDG-PET findings.

Parameters	MACE (*n =* 51)	No MACE (*n =* 69)	*p*-Value
Pattern			
Presence of mismatch only	6 (11.8)	8 (11.6)	1.00
Presence of match only	14 (27.5)	16 (23.2)	0.67
Presence of mismatch and match	31 (60.8)	45 (65.2)	0.70
Segment number			
Match	3.8 ± 2.7	3.4 ± 2.3	0.34
Mismatch	1.8 ± 1.6	2.3 ± 2.3	0.19
SRS	19.7 ± 9.2	19.9 ± 8.2	0.89
Summed FDG score	25.5 ± 12.2	27.9 ± 12.4	0.29
SRS if matched segments	14.3 ± 9.0	12.9 ± 8.0	0.41
SRS if mismatched segments	6.9 ± 4.6	8.9 ± 6.3	0.09
FDG score if matched segments	15.5 ± 9.5	13.6 ± 8.3	0.28
FDG score if mismatched segments	2.0 ± 2.0	2.6 ± 2.6	0.20
MRGlu values (umol/min/100 g)			
Global (exclude matched segments)	32.3 ± 23.4	22.3 ± 16.2	0.006
Low global MRGlu	19 (37.2)	41 (59.4)	0.01
CoV (exclude matched segments)	0.28 ± 0.15	0.35 ± 0.30	0.11
High CoV	30 (58.8)	56 (81.1)	0.007
Revascularization following imaging	12 (23.5)	14 (20.3)	0.82

Abbreviations: CoV, coefficient of variation; FDG, fluorodeoxyglucose; MACE, major adverse cardiovascular events; MRGlu, myocardial metabolic rate of glucose; SRS, summed resting score.

**Table 3:** Results of survival modeling.

	Adjusted HR	95% CI	*p*-Value
Global MRGlu (continuous)	1.01	1.002–1.02	0.009
Low global MRGlu (categorical)	0.48	0.27–0.87	0.01
High CoV (categorical)	0.65	0.35–1.21	0.18
Global MRGlu (continuous + categorical)			
Global MRGlu (continuous)	1.0	0.99–1.01	0.31
Low global MRGlu (categorical)	0.48	0.27–0.87	0.01
Interaction term (High CoV × Low global MRGlu)	0.35	0.18–0.68	0.001

Abbreviations: CI, confidence interval; CoV, coefficient of variation; HR, hazard ratio; MRGlu, myocardial metabolic rate of glucose.

## Data Availability

The data presented in this study are available on request from the corresponding author due to ethical restrictions.

## Supplementary Materials

The following supporting information can be downloaded at: https://www.mdpi.com/article/10.3390/diagnostics16142237/s1, Figure S1. Patient selection flowchart; Figure S2. Representative examples of the four metabolic patterns defined by combinations of high versus low MRGlu and high versus low CoV; Figure S3. Transplant-free survival according to expression levels of SLC2A1 (A), HK1 (B), IRS1 (C), and PKM (D); Table S1. Selected myocardial glucose-metabolism-related genes used for external transcriptomic analysis.

## Abbreviations

CI	confidence interval
CoV	coefficient of variation
DCM	dilated cardiomyopathy
FDG	^18^F-fluorodeoxyglucose
HR	hazard ratio
LVEF	left ventricular ejection fraction
MACEs	major adverse cardiac events
MRGlu	myocardial metabolic rate of glucose
NF	non-failing donor controls
PET	positron emission tomography
SPECT	single-photon emission computed tomography
SRS	Summed rest scores
