# Supplementary material for: Prognostic Value of Dynamic FDG PET-Derived Myocardial Glucose Metabolism in Ischemic Cardiomyopathy with Supportive External Gene-Expression Analysis
Source: Diagnostics (Basel). 2026 Jul 17;16(14):2237. doi: 10.3390/diagnostics16142237 (PMC13407970; doi:10.3390/diagnostics16142237)
Supplement: Supplementary file 1 [file diagnostics-16-02237-s001.zip › diagnostics-4371973-supplementary.pdf]

Supplementary Material

Prognostic Value of Dynamic FDG PET–Derived Myocardial Glucose Metabolism in Ischemic Cardiomyopathy with Supportive External Gene-Expression Analysis

Kuan-Yin Ko, Shan-Ying Wang, Hao-Yuan Tsai, Chien-Lin Lee, Jung-Cheng Hsu, Chung-Ming Tu, Yu-Chien Shiau, Kuan-Ming Chiu, Wen-Pin Chen, and Yen-Wen Wu

Corresponding author

Yen-Wen Wu, MD, PhD

Division of Cardiology, Cardiovascular Medical Center, Far Eastern Memorial Hospital

No. 21, Sec. 2, Nanya S. Rd., Banciao Dist., New Taipei City 220, Taiwan

E-mail: wuyw0502@gmail.com

Tel: +886-89667000 ext. 1090; Fax: +886-2-7728-2378

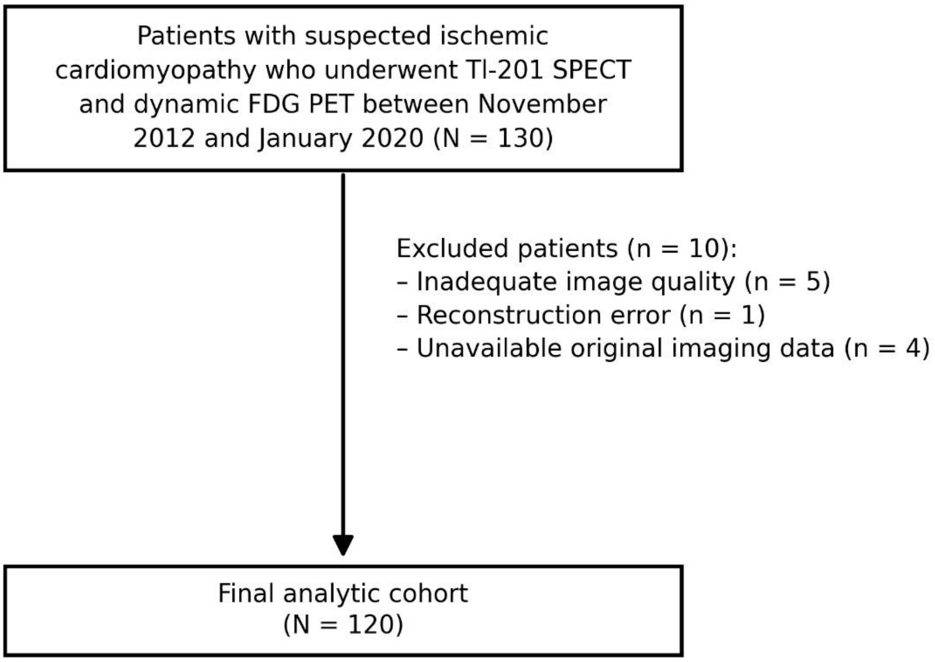

Figure S1. Patient selection flowchart

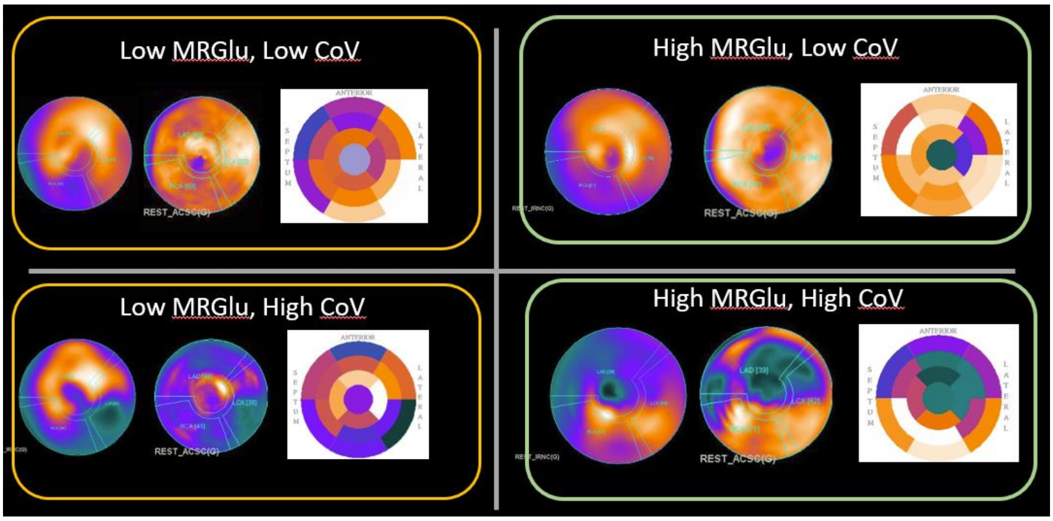

Figure S2. Representative examples of the four metabolic patterns defined by combinations of high versus low MRGlu and high versus low CoV.

Abbreviations: CoV, coefficient of variation; MRGlu, myocardial metabolic rate of glucose

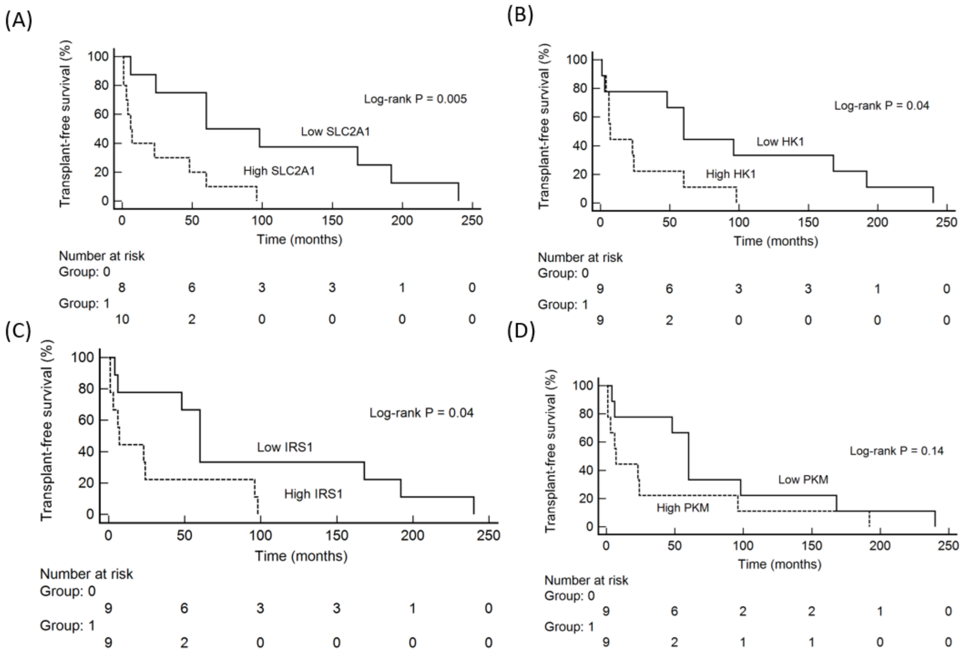

Figure S3. Transplant-free survival according to expression levels of SLC2A1 (A), HK1 (B), IRS1 (C), and PKM (D).

Abbreviations: HK1, hexokinase 1; IRS1, insulin receptor substrate 1; PKM, pyruvate kinase M1/2; SLC2A1, solute carrier family 2 member 1 (GLUT1)

Table S1. Selected myocardial glucose-metabolism–related genes used for external transcriptomic analysis

Gene	Pathway	Established role	Ref.
AKT1	Insulin signaling	Key downstream kinase in the insulin/PI3K pathway; promotes cardiac glucose uptake and utilization, including GLUT4 trafficking.	23
HK1	Glycolysis	Mitochondria-associated hexokinase that phosphorylates glucose/FDG and supports glycolytic flux, particularly in neonatal or high-glycolytic myocardium.	24
HK2	Glycolysis	Major adult cardiac hexokinase; couples glucose phosphorylation to mitochondrial metabolism and contributes to mitochondrial cardioprotection.	25
INSR	Insulin signaling	Insulin receptor that initiates IRS/PI3K/AKT signaling, regulating cardiac glucose uptake and substrate utilization.	23
IRS1	Insulin signaling	Key adaptor linking insulin receptor activation to PI3K/AKT signaling; impaired IRS1/IRS2 signaling contributes to cardiac insulin resistance and heart failure.	26
PFKM	glycolysis	Muscle phosphofructokinase isoform catalyzing the committed glycolytic step; deficiency impairs glycolytic flux.	27
PKM	glycolysis	Encodes M-type pyruvate kinase isoforms that catalyze the final ATP-generating step of glycolysis; PKM2 also regulates cardiac injury and regenerative pathways.	28
PRKAA1	AMPK / energy sensing	Catalytic AMPKα1 subunit involved in energy-stress signaling; AMPK activation supports glucose uptake and glycolytic adaptation during ischemia or energy depletion.	29
SLC2A1 (*GLUT1)*	Glucose transport	Basal cardiac glucose transporter; can be recruited to the sarcolemma during insulin stimulation and ischemia, supporting stress-related glucose uptake.	30
SLC2A4 (*GLUT4)*	Glucose transport / insulin signaling	Major insulin-responsive cardiac glucose transporter; cardiac GLUT4 deletion abolishes insulin-stimulated glucose uptake and induces compensatory GLUT1 upregulation.	31
